# Human beta defensin (HBD) gene copy number affects HBD2 protein levels: impact on cervical bactericidal immunity in pregnancy

**DOI:** 10.1038/s41431-017-0061-7

**Published:** 2018-01-24

**Authors:** Catherine P. James, Mona Bajaj-Elliott, Razan Abujaber, Frida Forya, Nigel Klein, Anna L. David, Edward J. Hollox, Donald M. Peebles

**Affiliations:** 1 UCL Institute for Women’s Health, 86-96 Chenies Mews, London, WC1E 6HX UK; 2 UCL Great Ormond Street Institute of Child Health, 30 Guilford Street, London, WC1N 1EH UK; 3 Department of Genetics and Genome Biology, University of Leicester, Leicester, LE1 7RH UK

## Abstract

Human beta defensin 2 (HBD2) is an endogenous mucosal antimicrobial peptide (AMP) upregulated during infection and inflammation. HBD2 is encoded by the *DEFB4* gene, which exhibits extensive copy number variation. Previous studies have demonstrated a relationship between HBD copy number and serum HBD2 protein levels; however, our current understanding of the influence of copy number on mucosal AMP function remains limited. This study explores the relationship between HBD copy number, cervicovaginal HBD2 protein levels and antimicrobial activity in 203 women with risk factors for preterm birth. We provide evidence that suggests HBD copy number modulates cervical antimicrobial immunity.

## Introduction

Human beta defensin 2 (HBD2) is an endogenous mucosal antimicrobial peptide (AMP) upregulated during infection and inflammation. *DEFB4*—the gene encoding HBD2—is on a 322 kb region of chromosome 8p23.1. This locus exhibits extensive copy number variation (CNV), with diploid copy numbers varying between 1 and 12 [1, 2]. This region is designated the beta defensin CNV (HBD CNV) and encompasses two loci 5 Mb apart, separated by a polymorphically inverted single copy sequence [3].

Previous studies indicate that *DEFB4* copy number correlates with serum HBD2 protein levels in healthy volunteers [4, 5]. Higher HBD copy number is associated with an increased risk of psoriasis, although the correlation between HBD copy number and serum HBD2 is not seen in psoriasis patients with active disease [6]. This is likely to be due in part to the cytokine-like properties of HBDs and may also indicate an inappropriate inflammatory response [6]. Indeed, in psoriasis patients with active disease HBD2 levels correlate with disease activity [4].

The fundamental biological question of how HBD copy number affects protein levels and antimicrobial activity on mucosal surfaces remains unanswered. This may have clinical implications in conditions where mucosal antimicrobial immunity may be a determinant between health and disease.

Preterm birth (PTB; delivery before 37 completed weeks of gestation) carries a high rate of perinatal mortality and morbidity. Chorioamnionitis (infection of the fetal membranes) is associated with 40–70% of cases [7]. In uncomplicated pregnancies, the sterile intrauterine cavity [8] is separated from the microbe-rich vagina by the endocervical canal. The cervical mucus plug (CMP) fills the cervical canal during pregnancy and has an antimicrobial barrier function [9], preventing ascending migration of bacteria from the vagina. The anionic CMP mucin skeleton allows the retention of positively charged molecules, including HBDs. We hypothesised that HBD copy number may modulate cervical HBD2 expression and hence the bactericidal capacity of the CMP.

## Methods

### Clinic population

Women of white European ancestry attending the University College London Hospital (UCLH) PTB clinic (PTBC, February 2010–May 2014) consented to the study. Analyses were restricted to this group (the largest group of PTBC attendees) to avoid the bias introduced by sampling a multiethnic group. The Joint UCL/UCLH Committees on the Ethics of Human Research approved the study (REC reference: 09/H0714/66). Six of the 209 women recruited started treatment with progesterone before samples were collected and were therefore excluded from the study. Median gestational age at recruitment was 15 weeks 2 days (range 8 + 5–27 + 3).

### Sample collection

At speculum examination, a liquid-based cytology cytobrush (Cellpath) was used to collect a cervical sample by two 360° sweeps of the cervical canal and snap frozen on dry ice. Upon thawing, 1.4 ml of 10 mM sterile HEPES buffer (Sigma-Aldrich) was added. After vortexing, the fluid was aspirated and 250 μl was removed for DNA extraction. The remainder was centrifuged (13,000 rpm, 15 min) and the supernatants were stored at −80 °C for protein and antimicrobial assays.

### Bacterial culture

*Escherichia coli* (strain ATCC25922) were grown on LB agar (Sigma-Aldrich) plates (37 °C, 5% CO_2_ for 24 h). Single colonies were suspended in LB broth (Sigma-Aldrich) and incubated at 37 °C (220 rpm) until mid-logarithmic phase was achieved.

### DNA extraction

DNA was extracted (QIAmp DNA Kit, Qiagen) according to the manufacturer’s instructions with an additional ribolysing step. Briefly, samples (200 μl) were incubated at 56 °C for 10 min with 20 μl Proteinase K, 1/6 vial Ribolyser resin (MP Biomedical, Santa Ana, California, USA) and 200 μl Buffer AL. The tubes were then ribolysed in a TissueLyser LT (Qiagen) at 50 m/s for 1 min and centrifuged (8000 rpm, 1 min). In all, 200 μl 100% ethanol was added and DNA extraction was completed according to the protocol.

### Beta defensin copy number assays

Beta defensin copy number was determined by triplex paralogue ratio test (PRT) as described [10, 11]. PCR products from each assay were combined and resolved by capillary electrophoresis (ABI 3130xl Genetic Analyzer, Applied Biosystems) with a Map Marker 400 X-Rhodamine ladder (Bioventures, Murfreesboro, Tennessee, USA). Peak area was determined using the ABI GeneMapper software (Applied Biosystems). Peak areas from samples were calibrated against reference DNA samples with known copy number. Data were first normalised by linear regression of the reference sample ratios against their expected integer copy numbers. The resulting regression equation was then used to adjust all other PRT data for each of the two dyes for the 107A and the HSPD21 assays. Finally, all three PRT copy number estimates were pooled and a maximum-likelihood calculation was used to determine the integer value that most accurately corresponded to the observed data. Copy number typing was successful in 95.6% (*n* = 194) samples and ranged from 1 to 8 with a modal copy number of 4, consistent with previous studies [12].

### Total protein assay and enzyme-linked immunosorbent assay (ELISA)

Filter-sterilised bovine serum albumin (Sigma-Aldrich) served as a standard. Coomassie reagent (Thermoscientific, Paisley, UK) was used according to the manufacturer’s instructions. Cervicovaginal fluid HBD2 levels were determined by ELISA (Peprotech ELISA Development Kit, according to the manufacturer’s instructions). HBD2 protein was undetectable in 25 samples; the lower limit of detection (16 pg/ml) divided by the square root of 2 was substituted [13].

### Antimicrobial assays

Exponential phase *E. coli* (ATCC25922, 50 μl, 10^5^ colony-forming units (cfu)/ml) were added to each cervical brush elution sample (50 μl, 37 °C) for 30 min. Bacteria survival in 10 mM sterile HEPES buffer for 30 min served as control. Postincubation, 20 μl of test was added to 180 μl sterile phosphate-buffered saline to terminate HBD-related killing activity. In all, 20 μl of the resultant mixture was inoculated into sterile prewarmed LG broth (180 μl, Sigma-Aldrich) followed by overnight incubation at 37 °C at 220 rpm. Bacterial quantification (cfu/ml) was measured at 600 nm in triplicate [14]. Percentage (%) of kill was calculated by considering the number of bacteria (cfu) in HEPES buffer as 100%. The value was then normalised to the total sample protein concentration.

### Analysis

Correlations were analysed pairwise between variables using the non-parametric Spearman rank correlation, implemented in GraphPad. Scatterplot matrices were plotted using R and show samples where values for all measured variables were available.

## Results and Discussion

**Fig. 1:**
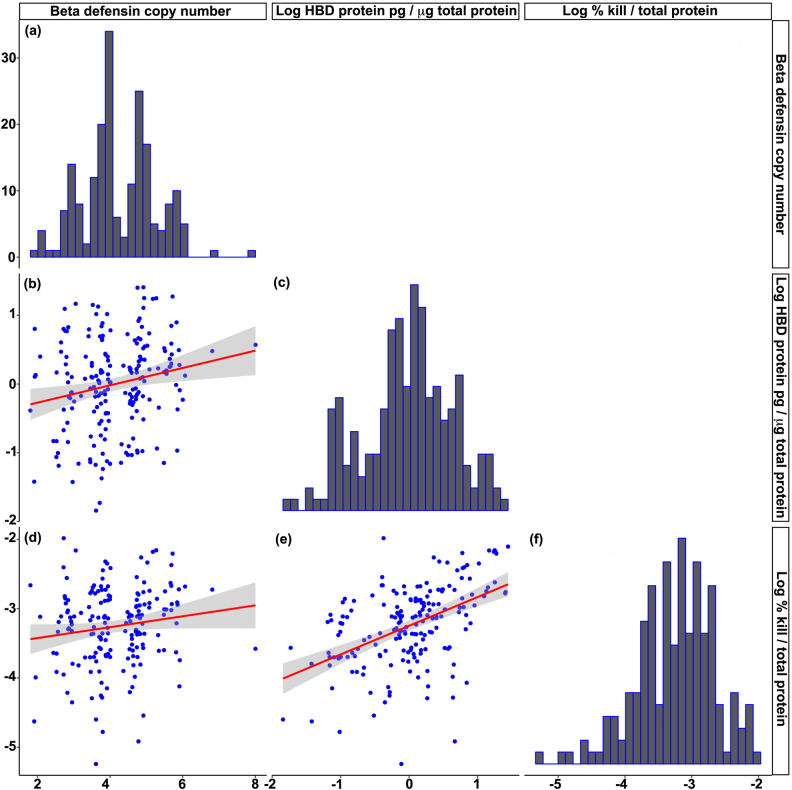
Beta defensin copy number, HBD2 protein expression and cervical antimicrobial activity. HBD2 protein concentration and antimicrobial activity (%kill/total protein) are presented on logarithmic scales. Beta defensin copy numbers are displayed as weighted mean PRT copy numbers. Red lines and grey-shaded regions on scatterplots represent regression lines and 95% confidence intervals. Copy numbers were determined by triplex PRT and protein levels were determined by ELISA. Histograms **a**, **c** and **f** display the distribution of values for beta defensin copy number, HBD2 protein concentration and antimicrobial activity, respectively. A significant positive correlation was observed between copy number and HBD2 protein levels (Plot **b**; Spearman *r* = 0.21, *p* = 0.0032). A weak but significant correlation was observed between copy number and antimicrobial activity (Plot **d** Spearman *r* = 0.17, *p* = 0.028. There was a strong correlation between HBD2 protein level and antimicrobial activity (Plot **e**; Spearman *r* = 0.49, *p* < 0.0001)

**Fig. 2:**
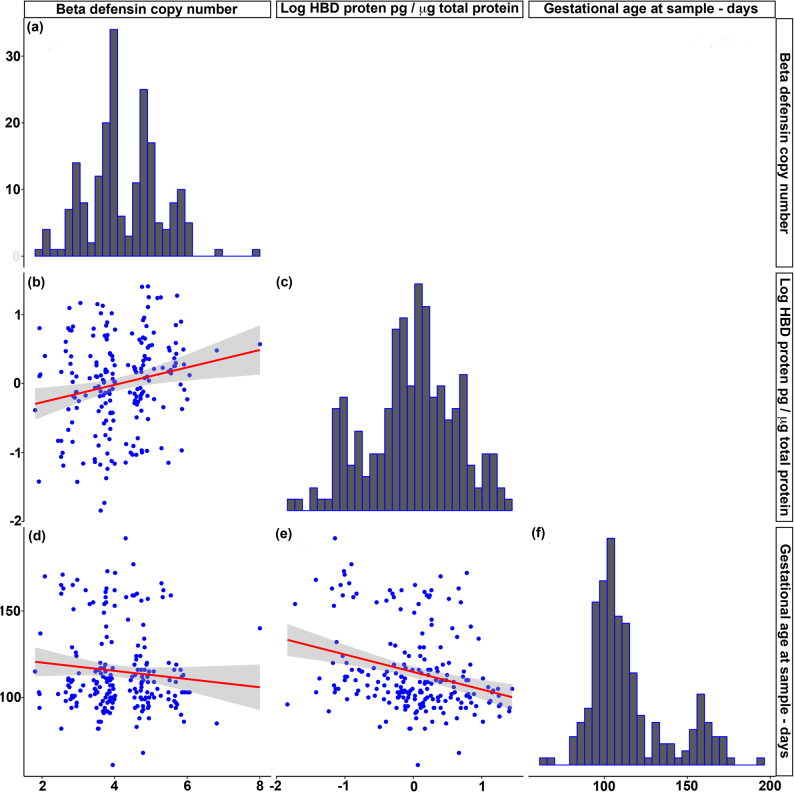
Beta defensin copy number, HBD2 protein expression and gestational age at sample collection. HBD2 protein concentration is presented on a logarithmic scale. Beta defensin copy numbers are displayed as weighted mean PRT copy numbers. Red lines and grey-shaded regions on scatterplots represent regression lines and 95% confidence intervals. Copy numbers were determined by triplex PRT and protein levels were determined by ELISA. Histograms **a**, **c** and **f** display the distribution of values for beta defensin copy number, HBD2 protein concentration and gestational age at sampling (in days), respectively. HBD2 protein levels decreased significantly with increasing gestational age at sample collection (Plot **e** Spearman *r* = −0.25, *p* = 0.0003); there was no correlation between copy number and gestational age at sample collection (Plot **d**). (Plots **b** and **c** are as described in Fig. 1)

Cervicovaginal HBD2 concentration across the cohort had a median of 1.2 pg/μg total protein (interquartile range (IQR) 0.44–3.0) and showed a positive correlation with HBD copy number (Spearman *r* = 0.21, *p* = 0.0032; Fig. 1). HBD2 concentration also displayed a negative correlation with gestational age at sample collection (Spearman *r* = −0.25, *p* = 0.0003, Fig. 2). However, there was no correlation between gestational age at collection and copy number (Fig. 2), so it is unlikely that the association between gestational age and protein concentration is influencing the relationship between copy number and protein concentration. We also evaluated cervicovaginal HBD3 concentration. There was no correlation between HBD copy number and cervicovaginal HBD3 concentration, despite *DEFB103* annotating to the HBD CNV (supplementary Fig. 1). This may be related to the common *DEFB103* promoter single-nucleotide polymorphism (SNP) rs273902, which has a clear effect on HBD3 mRNA expression [12]; combination of copy number and SNP genotype may therefore be more useful to evaluate the effects of each on protein expression.

We assessed the functional antimicrobial activity of cervicovaginal secretions by exposure to live bacteria. Across the cohort, the median killing activity was 0.049 %kill/μg total protein (IQR 0.015–0.13). There was a strong correlation between cervicovaginal antimicrobial activity and HBD2 protein levels (Spearman *r* = 0.49, *p* < 0.0001; Fig. 1). No single bacterial species is known to cause PTB, and HBD2 has an antimicrobial spectrum that includes a range of both Gram-positive and -negative bacteria and fungi [15]. *E. coli* was chosen as a model organism as it has been well described as susceptible to HBD2 in vitro [16]. A single model organism was chosen due to limited clinical sample volume.

As we observed a clear relationship between copy number and HBD2 protein levels, we hypothesised that there may be a direct correlation between copy number and antimicrobial activity. There was a weak but significant correlation between HBD copy number and cervicovaginal antimicrobial activity (Spearman *r* = 0.17, *p* = 0.028, Fig. 1).

Initial analyses were performed without considering pregnancy outcome. It is possible that women delivering preterm may have different AMP gene regulation at the cervix than women delivering at term. We excluded women who had treatment to prevent PTB after sample collection (cervical cerclage, *n* = 20 and/or progesterone, *n* = 2) and those who had an early miscarriage (*n* = 1) or termination of pregnancy (*n* = 1). Copy numbers were available for 153 out of the 160 women who delivered at term and all 14 women who delivered preterm. In term deliveries, the correlations between copy number and HBD2 protein levels and cervicovaginal antimicrobial activity remained (supplementary Fig. 2). There was no correlation between HBD copy number and either HBD2 protein or antimicrobial activity in women who delivered preterm. Interestingly, the correlation between HBD2 protein levels and cervicovaginal antimicrobial activity was also evident in samples from women who delivered preterm (Spearman *r* = 0.62, *p* = 0.027) (supplementary Fig. 3). It is possible that the absent correlation between HBD copy number and HBD2 protein and cervical antimicrobial activity in the preterm group is related to the study power; a larger cohort of women delivering preterm would be needed to determine this. If the lack of correlation is maintained with a larger cohort, it is possible that this may be due to the impact of the infectious–inflammatory axis as observed in patients with active psoriasis. There were no differences between modal copy number, HBD2 protein levels or cervical antimicrobial activity between women delivering preterm and those delivering at term.

This study is the first to investigate and describe the relationship between *DEFB4* copy number and HBD2 expression at the cervical mucosa in pregnancy. We report increased antimicrobial activity in samples with higher HBD2 concentrations. Finally, these data suggest an underlying relationship between beta defensin copy number and the antimicrobial activity of cervical secretions, raising the hypothesis that HBD copy number and the corresponding antimicrobial axis plays a critical role in mucosal health.

## Electronic supplementary material

Supplemental Figure 1

Supplemental Figure 2

Supplemental Figure 3
