# Supplementary material for: Human beta defensin (HBD) gene copy number affects HBD2 protein levels: impact on cervical bactericidal immunity in pregnancy
Source: Eur J Hum Genet. 2018 Jan 24;26(3):434–9. doi: 10.1038/s41431-017-0061-7 (PMC5831986; doi:10.1038/s41431-017-0061-7)
Supplement: Supplementary file 1 [file 41431_2017_61_MOESM1_ESM.pdf]

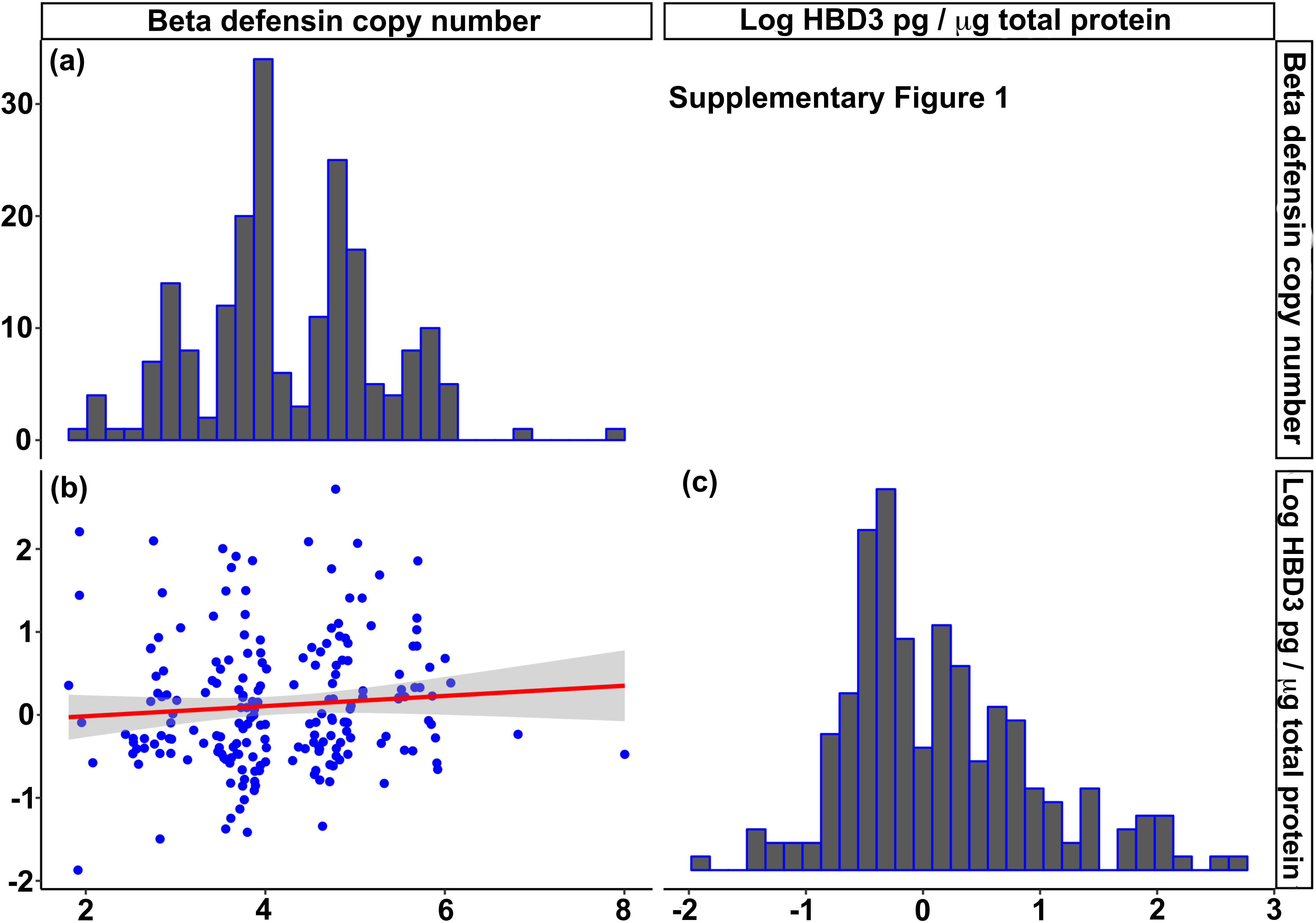

Beta defensin copy number
Log HBD3 pg / μg total protein
(a)
Supplementary Figure 1
Beta defensin copy number
(b)
(c)
Log HBD3 pg / μg total protein
30
20
10
0
2
1
0
-1
-2
2
4
6
8
-2
-1
0
1
2
3
