# Supplementary material for: Human beta defensin (HBD) gene copy number affects HBD2 protein levels: impact on cervical bactericidal immunity in pregnancy
Source: Eur J Hum Genet. 2018 Jan 24;26(3):434–9. doi: 10.1038/s41431-017-0061-7 (PMC5831986; doi:10.1038/s41431-017-0061-7)
Supplement: Supplementary file 2 [file 41431_2017_61_MOESM2_ESM.pdf]

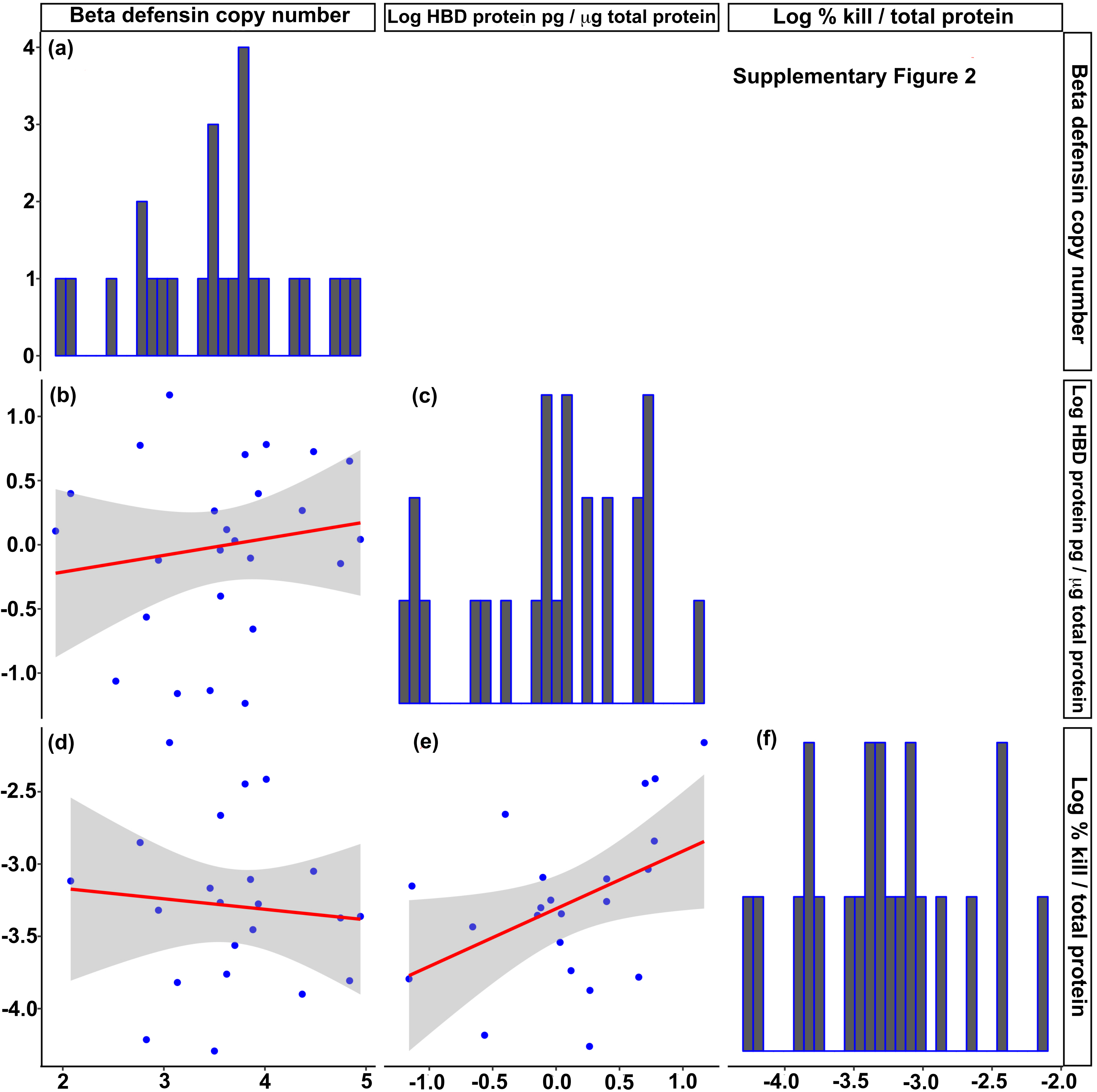

Beta defensin copy number
Log HBD protein pg / μg total protein
Log % kill / total protein
Supplementary Figure 2
(a)
(b)
(c)
(d)
(e)
(f)
Beta defensin copy number
Log HBD protein pg / μg total protein
Log % kill / total protein
